# Differential Effects of IL-17A and TNF-α on Osteoblastic Differentiation of Isolated Synoviocytes and on Bone Explants from Arthritis Patients

**DOI:** 10.3389/fimmu.2015.00151

**Published:** 2015-04-07

**Authors:** Bilal Osta, Jean-Paul Roux, Fabien Lavocat, Marlène Pierre, Ndieme Ndongo-Thiam, Georges Boivin, Pierre Miossec

**Affiliations:** ^1^Immunogenomics and Inflammation Research Unit EA 4130, Department of Clinical Immunology and Rheumatology, Edouard Herriot Hospital, University of Lyon 1, Lyon, France; ^2^UMR 1033, INSERM, UFR de Médecine Lyon Est, Lyon, France

**Keywords:** bone, osteoblastogenesis, synoviocytes, TNF-α, IL-17A, rheumatoid arthritis, osteoarthritis

## Abstract

**Objective:**

TNF-α and IL-17A act on fibroblast-like synoviocytes (FLS) and contribute to cytokine production, inflammation, and tissue destruction in rheumatoid arthritis (RA). The aim of this study was to compare their effects on osteogenic differentiation of isolated FLS and on whole bone explants from RA and osteoarthritis (OA) patients.

**Methods:**

Fibroblast-like synoviocytes and bone explants were cultured in the presence or absence of TNF-α and/or IL-17A. Mineralization of extracellular matrix of FLS was measured by alizarin red and alkaline phosphatase activity (ALP). mRNA expression was analyzed by qRT-PCR for Wnt5a, BMP2, and RUNX2, key genes associated with osteogenesis. IL-6 and IL-8 levels were measured by enzyme-linked immunosorbent assays. Bone explant structure was quantified by histomorphometry.

**Results:**

In isolated OA and RA FLS, the combination of TNF-α and IL-17A induced matrix mineralization, increased ALP activity and expression of the osteogenesis-associated genes Wnt5a, BMP2, and Runx2, indicating an osteogenic differentiation. Wnt5a levels increased with TNF-α alone and in combination with IL-17A. BMP2 expression decreased with IL-17A and TNF-α after 12 h with OA FLS and 24 h with RA FLS. Runx2 expression decreased only with combination of TNF-α and IL-17A in OA FLS and with cytokines alone and combined in RA FLS. IL-6 and IL-8 production increased with IL-17A and/or TNF-α in both FLS and bone samples, especially from RA. Treatment of bone explants with cytokine combination increased ALP in OA but not RA samples. A decrease in bone volume was seen with cytokine combination, especially with RA explants.

**Conclusion:**

Differences were observed for the effects of IL-17A and TNF-α on osteogenic differentiation. In isolated FLS, increased osteoblastogenesis was observed, contrasting with the inhibitory effect in whole bone, specifically in RA. The net effect of IL-17A and TNF-α appears to depend on the disease state and the presence of other cells.

## Introduction

Arthritis diseases are associated with various local bone changes. Rheumatoid arthritis (RA) is characterized by destruction of bone, and defective repair leading to rapid joint destruction. Osteoarthritis (OA) is characterized by simultaneous bone destruction and osteophyte formation, leading to a lower speed of joint destruction. Fibroblast-like synoviocytes (FLS) are the predominating cells in the synovium, and play a central role in defining the stromal environment within arthritic bone diseases. *In vitro* studies have shown that FLS cultured in osteogenic medium undergo osteogenic differentiation with a significant increase in alkaline phosphatase and calcium deposits (1, 2). However, the effects of inflammation and of cytokines in the context are unknown.

Bone homeostasis is maintained by the balance of bone resorption, mediated by osteoclasts (OCs) (3) and bone formation, mediated by osteoblasts (OBs) (4). Most of the *in vitro* studies on bone homeostasis usually covered only one aspect of this balance, either bone formation or destruction, using isolated cell types. However, a complex system exists *in vivo* with interactions between bone cells (OCs, OBs, osteocytes), bone marrow cells, and a complex network of cytokines, hormones, and other signaling molecules under the influence of mechanical stimuli (5–9).

In the context of arthritic diseases, this occurs through local production of pro-inflammatory cytokines, like TNF-α and IL-17A, small molecule mediators of inflammation, and proteolytic enzymes that degrade the extracellular matrix leading to bone and cartilage destruction (10–14). Most of the *ex vivo* models have suggested inhibition of TNF-α and IL-17A to control bone destruction (15–19). However, recent studies have shown that TNF-α and IL-17A may induce bone formation *in vitro* (20–23).

We have previously studied the effects of IL-17A and TNF-α on the *in vitro* osteogenic differentiation of bone marrow-derived mesenchymal cells from normal individuals (23). In the context of local arthritis, it has been well established that chronic inflammation induces overtime various epigenetic changes in mesenchymal cells. When comparing RA to OA FLS, one major effect of IL-17 was the acquisition of a defective apoptosis in RA FLS, contributing chronicity (24, 25). To study joint-derived mesenchymal cells, which have been exposed to this long-lasting inflammatory stress, we used isolated OA and RA FLS as they are easy to obtain and compared their response to IL-17A and TNF-α regarding osteogenic differentiation and cytokine production. We then compared the effects of these cytokines on isolated cells and on *ex vivo* bone explants from OA and RA patients. Matrix mineralization, alkaline phosphatase (ALP), cytokines, and key regulatory genes of osteogenesis were examined for the *in vitro* FLS model. ALP, cytokines, and histomorphometry were used for *ex vivo* studies.

## Materials and Methods

### Patients

Bone explants were obtained from the tibia and femoral head from 13 patients undergoing orthopedic surgery for joint replacement (3 RA and 10 OA; 4 males/9 females; age 62.0 ± 14.3 years). RA and OA patients fulfilled the American College of Rheumatology criteria for RA and OA (26, 27). Each individual signed an informed consent and the protocol was approved by the committee for protection of persons participating in biomedical research.

### Preparation and culture of bone explants and synovial cells

Bone samples were obtained under sterile conditions and immediately placed in phosphate buffer saline (PBS) containing antibiotics. Sample were cut into pieces of ~500 mm^3^, and cultured in complete αMEM medium (Lonza, Verviers, Belgium) supplemented with 10% FBS (Hyclone, Thermo scientific, South America), 2 mM l-glutamine, 100 U/ml penicillin, streptomycin at 37°C in a humidified environment of 5% CO_2_, and 95% air. Cultures were performed in six well-plates (Falcon) with one piece of bone in a final volume of 8 ml, and in the presence or absence of 1 ng/ml TNF-α and/or 50 ng/ml IL-17A (R&D systems, Lille, France). Supernatants were harvested after 7 days of culture, and the remaining bone samples were used for histomorphometry.

Fibroblast-like synoviocytes were obtained from the synovial tissue of hips or knees from three RA and three OA patients. FLS were isolated by enzyme digestion and cultured at 37°C in DMEM medium (Eurobio, Courtaboeuf, France) supplemented with 10% fetal bovine serum (Thermo scientific, Saint Aubin, France), 2 mM l-glutamine, 100 U/ml penicillin, streptomycin. FLS were then used for osteogenic differentiation (23).

### Mineralization assay

Cells were washed twice with PBS, fixed with 70% cooled ethanol for 1 h, and washed with water. Cells were stained for 20 min at ambient temperature with alizarin red (pH: 4.2, 40 min, Sigma, Saint Quentin-Fallavier, France) and examined under light microscope. The red color referred to calcium deposit was quantified. The alizarin scale is defined as the average of clusters counted under microscope divided by 10 to simplify the scale.

### Alkaline phosphatase assay

Wells were washed twice to remove any dead cell, then supernatants of cell lysates were collected, and protein content was determined using the Bradford protein assay (Sigma). Ten microliters from the supernatants were mixed with 20 μl of MUP, used as a substrate (Abcam, Paris, France) in a 96-well plate, and incubated at room temperature for 30 min. Fluorescence intensity was measured at excitation/emission 360/440 nm. The ALP activity was normalized to protein content and expressed as U/μg protein.

### Quantitative RT-PCR analysis

RNA was purified using RNeasy kits (Qiagen, Les Ulis, France). The concentration of RNA was quantified by spectrophotometry (SmartSpec™ 3000, Biorad, Hercules, CA, USA). Total RNA (500 ng) was reverse transcribed with the QuantiTec Reverse Transcription (Qiagen Kit) into cDNA. PCR amplification was performed on a LightCycler (Roche Diagnostics, Switzerland) using Fast-Start™ DNA Master SYBR Green I real-time PCR kit (Roche Molecular Biochemicals, Switzerland). The expression of the genes was normalized to the expression of human cyclophilin B (CPB) (5′tgtggtgtttggcaaagttc3′; 3′gtttatcccggctgtctgtc5′) (Qiagen). The list of primers (Qiagen) is as follows: BMP2 (5′ccaccatgaagaatctttgga3′; 3′gagttggctgttgcaggttt5′), RUNX2 (5′gtggacgaggcaagagttt3′; 3′tggggtctgtaatctgactc5′), Wnt5a (5′attcttggtggtcgctaggt3′; 3′accttcgatgtcggaattgat5′).

### Enzyme-linked immunosorbent assays

Concentrations of IL-6 and IL-8 were evaluated with commercial enzyme-linked immunosorbent assays (ELISA) kits, according to the manufacturer’s instructions (R&D systems, Lille, France).

### Bone histomorphometry

This study was performed as previously described (28, 29). Bone pieces were fixed in cold 4.5% formalin (pH 7.4) and embedded in methylmethacrylate (Merck, Fribourg, Germany) at −20°C. Sections of 8 μm were prepared with a microtome (Polycut E, Reichert-Jung, Germany) and stained with Solochrome cyanin R, and Goldner’s (all reagents from Merck). Histomorphometric parameters were measured with automatic image analyzer at low power field (×1) (Imager A1, Axio, Zeiss, Germany).

### Statistical analysis

Analysis was performed using a Wilcoxon test from Graphpad Prism. *p* Values were determined taking into account the number of samples from different patient and the number of replicates per assay. *p* Values of <0.05 were considered significant.

## Results

### TNF-α and IL-17A increased mineralization of the extracellular matrix in isolated synoviocytes

**Figure 1:**
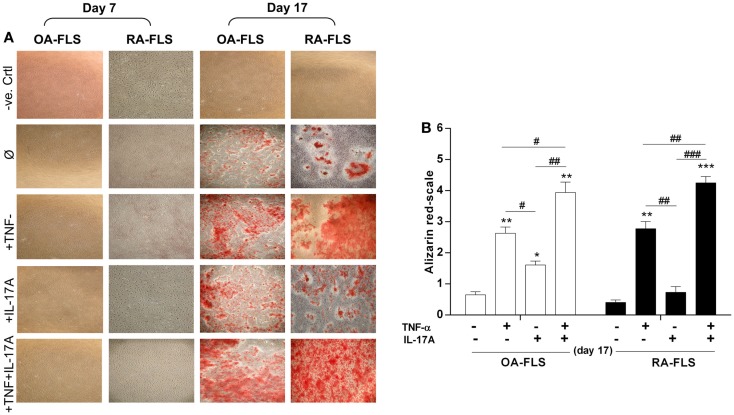
**Effects of IL-17A and/or TNF-α on extracellular matrix of synoviocytes**. FLS were cultured at a density 5 × 10^3^ cell/cm^2^. **(A)** In the absence (column 1, negative control as basic DMEM medium without any addition) or presence (column 2) of osteogenic factors. TNF-α 1 ng/ml (column 3) or IL-17A 50 ng/ml (column 4), or both (column 5) were added or not to cultures. After 7, 14, and 17 days, cells were stained with alizarin red and **(B)** calcium deposits were quantified and analyzed for each condition using the Wilcoxon test *p < 0.05, **p < 0.005, ***p < 0.0005 vs. induction medium alone, ^#^*p* < 0.05, ^##^*p* < 0.005 TNF-α alone vs. IL-17A and IL-17A + TNF-α, ^###^*p* < 0.0005 IL-17A vs. IL-17A + TNF-α. The bars of each graph represent mean + SEM.

Mineralization of the extracellular matrix is one of the key bone formation markers and is influenced by cytokines (30). To evaluate the effects of TNF-α and/or IL-17A on matrix mineralization, FLS from OA and RA patients were cultured for 17 days in osteogenic medium with and without cytokines. Alizarin red staining was used to visualize mineralization. As shown in Figure 1A, row 2, culture of FLS with osteogenic factors alone induced a weak matrix mineralization, which appeared at day 17 in both cell types. In OA-FLS, both TNF-α and IL-17A enhanced significantly this mineralization at day 17 (Figure 1A, row 3 and 4; Figure 1B). In RA FLS, TNF-α but not IL-17A alone enhanced matrix mineralization (Figure 1A, row 4; Figure 1B). Moreover, the combination of TNF-α and IL-17A highly enhanced the mineralization in both cell types (Figure 1A, column 5). Thus, these results show that TNF-α alone enhanced bone mineralization, while a potentiation was observed upon combination of TNF-α and IL-17A.

### IL-17A and TNF-α increased ALP activity *in vitro* but not *ex vivo*

**Figure 2:**
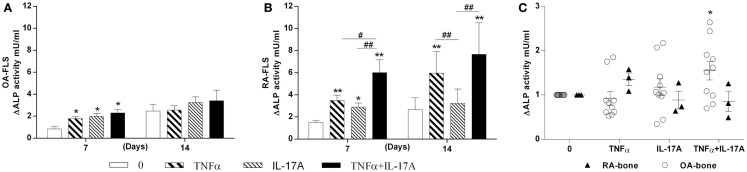
**Effects of IL-17A and/or TNF-α on alkaline phosphatase activity**. **(A)** OA and **(B)** RA FLS were cultured at a density 5 × 10^3^ cell/cm^2^ in osteogenic medium in the absence (0) or presence of TNF-α 1 ng/ml and/or IL-17A 50 ng/ml for 14 days. **(C)** Bone samples were cultured in six well-plates for 7 days. Supernatants were collected for ALP measurements by fluorometry. Results were analyzed using the Wilcoxon test. **p < 0.005 vs. osteogenic medium alone, ^#^*p* < 0.05, ^##^*p* < 0.005 TNF-α alone vs. IL-17A and IL-17A + TNF-α, ^##^*p* < 0.005 IL-17A vs. IL-17A + TNF-α. The bars of each graph represent mean + SEM.

Next we investigated the effects of TNF-α and IL-17A on ALP, an enzyme essential for bone mineralization (31). ALP activity was measured at day 7 and 14 (Figure 2). In OA and RA FLS, TNF-α and IL-17A alone or combined increased ALP activity at day 7 (Figures 2A,B). Day 14 shows no more difference between osteogenic medium alone and cytokines in OA FLS (Figure 2A), while in RA FLS, TNF-α alone or in combination to IL-17A showed a significant increase (6-fold with TNF-α and 7.3-fold with TNF-α + IL-17A vs. 2.2-fold without cytokine, **p < 0.005). The combination of both cytokines showed a more potent effect on RA than OA FLS.

In parallel, ALP was measured in the supernatants of OA and RA bone samples cultured for 7 days. TNF-α and IL-17A alone induced a non-significant increase in OA but not in RA samples. A significant increase was seen with the combination of both cytokines in OA but not RA samples (Figure 2C). RA bone samples appeared thus insensitive to the effect of cytokines. Therefore, these results showed that *in vitro*, IL-17A and TNF-α combination increased ALP in OA FLS, with increased sensitivity in RA FLS, while *ex vivo*, only OA bone samples showed a limited response.

### IL-17A and TNF-α increased osteogenic gene expression in FLS

To better understand the effects of TNF-α and IL-17A on osteogenic differentiation of FLS, mRNA expression levels of osteogenic genes like BMP2 (32), Wnt5a (33), and Runx2 (34) were measured at 6, 12, and 24 h.

**Figure 3:**
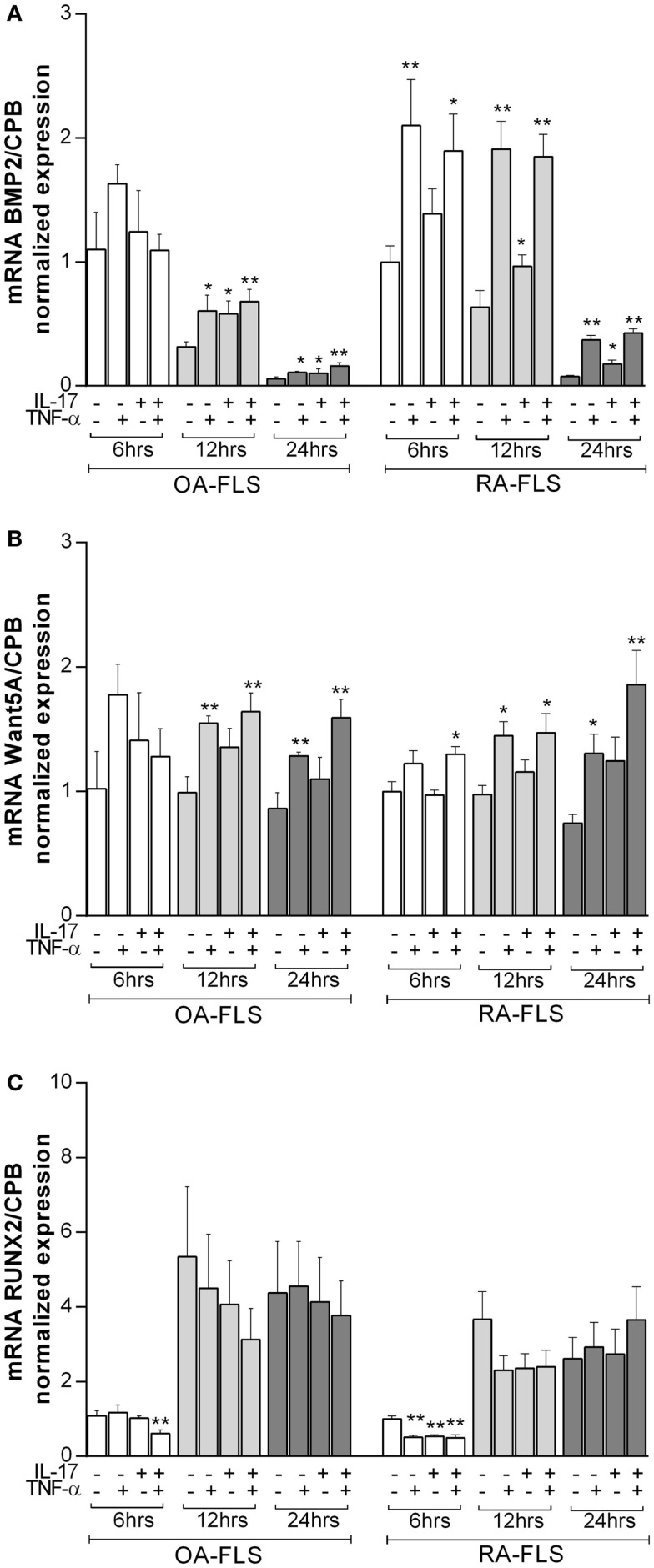
**Effects of TNF-α and IL-17A on osteogenic marker genes**. FLS were cultured in osteogenic medium after 1 day of plating, in the presence or absence of TNF-α 1 ng/ml and/or IL-17A 50 ng/ml for the indicated times. Osteogenic gene expression of **(A)** BMP2, **(B)** Wnt5a, and **(C)** Runx2 was measured by q-RT-PCR at 6, 12, and 24 h. Results were analyzed using the Wilcoxon test. *p < 0.05; **p < 0.005 vs. without cytokines. The bars of each graph represent mean + SEM.

Bone morphology protein 2 (BMP2) is known to induce OB differentiation (32). At 6 h, TNF-α alone and combined with IL-17A increased BMP2 expression levels in RA but not OA FLS. IL-17A alone had no effect. Compared to 6 h levels, BMP2 expression decreased over time in the presence of IL-17A and TNF-α in OA FLS (1 vs. 0.68 vs. 0.16-fold decrease at 6, 12, and 24 h, respectively), while it started decreasing at 24 h in RA FLS (1.8-fold with IL-17A + TNF-α at 6 and 12 h vs. 0.42-fold with TNF-α + IL-17A at 24 h) (Figure 3A). Moreover, this increase in BMP2 expression was more potent in RA than OA-FLS (1.8-fold increase with IL-17A + TNF-α compared to control without cytokines in RA FLS at 12 h vs. 0.68-fold in OA FLS, **p < 0.005).

Wnt5a plays an important role in the development of bone and induces osteoblastogenesis (33). Basal Wnt5a expression levels did not change over time. IL-17A alone had no significant effect on Wnt5a expression, while TNF-α alone or in combination with IL-17A induced a significant increase after 12 h in both OA and RA FLS (1.6-fold increase with IL-17A + TNF-α vs. 0.9-fold without cytokines in OA FLS, **p < 0.005; 1.4-fold increase with IL-17A + TNF-α vs. 0.9-fold without cytokines in RA FLS, *p < 0.05) (Figure 3B).

Runx2 is a key transcription factor for osteogenesis (34). Runx2 mRNA expression increased over time as early as 12 h in OA and RA FLS (1-fold without cytokines at 6 h vs. 5-fold at 12 h in OA FLS; 1-fold without cytokines at 6 h vs. 3.6-fold at 12 h in RA FLS) (Figure 3C), which is consistent with the ongoing osteogenic differentiation. Cytokine treatment decreased Runx2 expression in OA and RA FLS at early time points (0.6-fold with IL-17A + TNF-α vs. 1-fold without cytokines in OA FLS and 0.5-fold with IL-17A + TNF-α vs. 1-fold without cytokines in RA FLS at 6 h, **p < 0.005), whereas, at later time points, these cytokines had no effect on Runx2 expression.

Therefore, these results show that IL-17A and TNF-α combination induced significant changes in osteogenic gene markers of both FLS, specifically with RA FLS.

### Potentiation effects of IL-17A and TNF-α on IL-6 and IL-8 production *in vitro* and *ex vivo*

**Figure 4:**
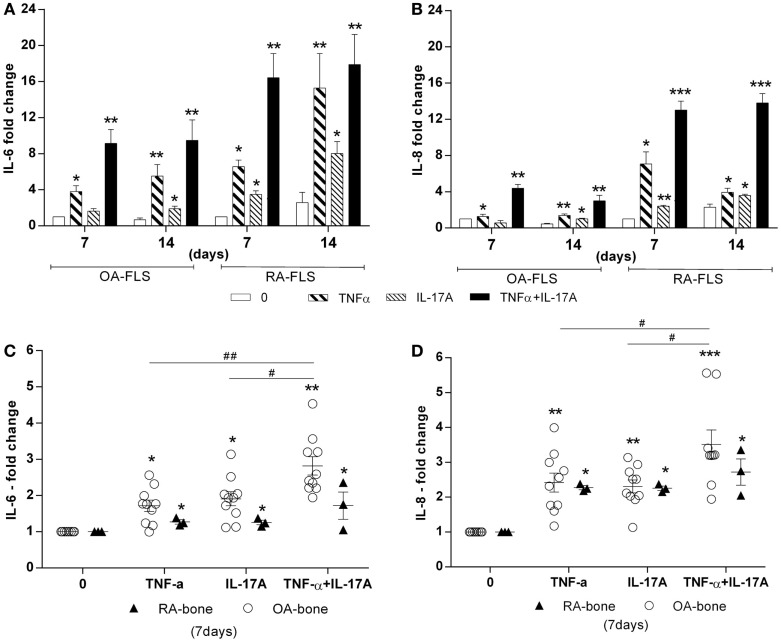
**Effects of TNF-α and IL-17A on IL-6 and IL-8 production**. **(A)** OA and **(B)** RA FLS were cultured in osteogenic medium for 14 days and bone samples **(C,D)** for 7 days, in the presence or absence of TNF-α 1 ng/ml and/or IL-17A 50 ng/ml. IL-6 **(C)** and IL-8 **(D)** concentrations in supernatants were quantified by ELISA. Results were analyzed using the Wilcoxon test. *p < 0.05, **p < 0.005 vs. osteogenic medium alone. The bars of each graph represent mean + SEM.

TNF-α and IL-17A are known to induce IL-6 and IL-8 production, with IL-6 being a key cytokine for osteoclastogenesis (35, 36). Treatment with IL-17A and TNF-α alone or combined for 7 and 14 days induced IL-6 and IL-8 production in both *in vitro* and *ex vivo* models, especially with IL-17A and TNF-α combination (Figure 4). *In vitro*, levels were higher in RA compared to OA FLS at both 7 and 14 days, for both IL-6 (9.4-fold with TNF-α + IL-17A vs. 0.6-fold without cytokines for OA FLS, **p < 0.005; 17-fold with TNF-α + IL-17A vs. 2.5-fold without cytokines for RA FLS, **p < 0.005), and IL-8 (3-fold with TNF-α + IL-17A vs. 0.4-fold without cytokines OA, **p < 0.005; 13.8-fold with TNF-α + IL-17A vs. 2.2-fold without cytokines RA, ***p < 0.0005) (Figures 4A,B).

*Ex vivo* results with bone samples showed changes in accordance with those obtained *in vitro*, where IL-17A and/or TNF-α increased significantly IL-6 and IL-8 production (Figures 4C,D). However, bone samples from RA patients showed reduced reactivity compared to bone samples from OA patients, possibly as a consequence of previous *in vivo* exposure to the same factors. Thus, these results showed that IL-17A and TNF-α exerted the same effect on IL-6 and IL-8 production in both *in vitro* and *ex vivo*, OA bone samples being more sensitive.

### Effects of IL-17A and TNF-α on BV/TV ratio

**Figure 5:**
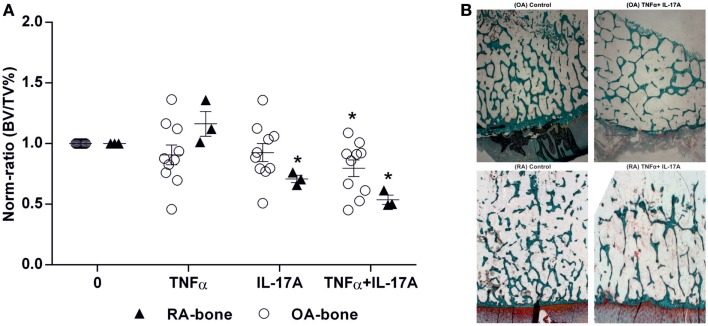
**Effects of IL-17A and/or TNF-α on BV/TV ratio**. Bone samples were cultured for 7 days in six well-plates, in the presence or absence of IL-17A (50 ng/ml) and TNF-α (1 ng/ml) or both, then bone samples were used for histomorphometric analysis. Bone volume over tissue volume (BV/TV) was measured with an automatic image analyzer at low power field. **(A)** Results were analyzed using the Wilcoxon test. *p < 0.05 vs. without cytokines. The bars of each graph represent mean + SEM. **(B)** Representative histomorphometric sections from OA and RA bone samples with and without the combination of IL-17A and TNF-α. Goldner’s staining shows trabecular bone in green (10× magnification).

The key parameter for bone destruction is the quantification of bone by histomorphometry. Bone volume over total volume (BV/TV) ratio was calculated after 7 days of culture in the presence of IL-17A and/or TNF-α. In RA bone explants, IL-17A alone or combined with TNF-α induced a significant decrease in BV/TV ratio (0.65-fold with IL-17A and 0.53-fold with IL-17A + TNF-α vs. 1-fold without cytokines, *p < 0.05) whereas in OA bone explants, only the combination of cytokines induced a significant decrease (0.79-fold with IL-17A + TNF-α vs. 1-fold without cytokines, *p < 0.05) (Figure 5A). Representative histomorphometric sections are shown in Figure 5B.

Thus, these results showed that TNF-α and IL-17A decreased BV/TV ratio, with RA samples being more sensitive to their destructive properties.

## Discussion

TNF-α is well known to induce bone degradation in arthritic diseases, and IL-17A is now considered a new interesting target since it shows synergy with TNF-α. The aim of this study was thus to evaluate the effects of these cytokines *in vitro* using isolated FLS and *ex vivo* using bone explants. Our results demonstrated that isolated OA and RA synoviocytes were induced toward osteogenic differentiation in the presence of the two cytokines via increasing matrix mineralization and effects on osteogenic genes such as BMP2, Wnt5a, and Runx2. On the other hand, the *ex vivo* model showed rather a shift toward bone degradation.

Differences between OA- and RA-FLS and explants were observed. RA-FLS were more sensitive to the effects of TNF-α and IL-17A on cytokine production, ALP production but not matrix formation. In RA bone samples, TNF-α and IL-17A induced less ALP and cytokine production and more bone matrix loss compared to OA samples.

The effect of TNF-α on whole bone destruction is in full agreement with large use of TNF-α inhibitors in RA, but not in OA. In addition, RA explants showed an increased sensitivity to cytokines. Opposite results were obtained when isolated FLS were studied for osteogenic differentiation. Since the two systems differ from their cell complexity, we concluded that the key difference was related to the presence or not of OCs, as the only cells able to resorb bone.

Regarding the key genes associated with osteogenesis, BMP2 expression was previously found to be increased in different cell types such as endothelial cells, synoviocytes, and chondrocytes in response to TNF-α (37, 38). This report extends these findings with the effects of TNF-α and IL-17A alone and combined on BMP2 expression in FLS during osteogenesis. Here, TNF-α and/or IL-17A induced early BMP2 expression with higher levels in RA, compared to OA FLS. Wnt5a also showed an increase in the presence of TNF-α alone or when combined to IL-17A in both OA and RA FLS. Runx2 is a key transcription factor for osteogenic differentiation (34). Indeed, in the presence of osteogenic medium alone, Runx2 mRNA expression increased over time in OA and RA FLS, which is consistent with the ongoing osteogenic differentiation. Conversely, this enhancing effect was inhibited in the presence of both cytokines again in OA and RA FLS.

Addition of exogenous cytokines had a predicted effect on endogenous production of other cytokines as part of an amplification mechanism. Numerous studies have described TNF-α as a dominate inducer of IL-6 and IL-8 in synovium and IL-17A shows enhancing and synergistic effects with TNF-α (39, 40). Mouse and human joint explant studies suggested that IL-17A alone can induce inflammatory response (17, 36). In this study, TNF-α and/or IL-17A induced IL-6 and IL-8 production in both isolated OA and RA FLS, with higher levels with RA FLS. These results indicate that the addition of TNF-α and IL-17A to isolated OA and RA FLS can induce local inflammation. IL-6 is a well-known OC activating factor acting as a soluble link between OBs and OCs. Combination of TNF-α and IL-17A specifically in RA FLS and explants would then activate matrix destructing OCs (41). IL-8 is a key chemotactic factor for neutrophils. Here, again the combination of TNF-α and IL-17A specifically in RA FLS and explants would then activate bone marrow progenitors leading to the maturation of activation of neutrophils (42). In turn, this would lead to bone edema, which is an early event at sites of bone destruction in RA (43).

These effects on isolated cells might not reflect the cell interactions that occur *in vivo* in bone. Indeed, in the *ex vivo* model, where OBs and OCs interact together and with bone marrow cells, important differences were noted. ALP increased in this *ex vivo* model with the combination of both cytokines in OA but not RA samples while *in vitro* ALP increased in both FLS, especially from RA. This already indicated a defect in bone formation in RA explants. Results of IL-6 and IL-8 production in bone explants coincide with those obtained *in vitro*, indicating local inflammation, leading to OC activation and bone edema. This was indeed confirmed by histomorphometric measurements of BV/TV ratio, which showed that the combination of TNF-α and IL-17A significantly decreased bone structure, specifically with RA samples.

The interaction between different cell types combining bone cells and bone marrow cells within the *ex vivo* model result in a different pattern of response to the TNF-α and IL-17A effects. This suggests that their net effects in arthritic diseases will depend on a balance between osteogenesis and osteoclastogenesis processes at different ratios. RA samples appear highly sensitive to the effects of TNF-α and IL-17A. These results are in line with several studies with RA-derived bone explants where inhibition of TNF-α decreased inflammation as measured by levels of IL-6 and bone resorption markers. These inhibitory effects were further enhanced with the combined blockade of IL-1 and IL-17A (18). In the same RA *ex vivo* model of bone resorption, IL-4-mediated inhibition of TNF-α level was associated with a 35% inhibition in bone resorption with IL-4 (16). On the other side, IL-17A enhanced bone resorption and decreased formation in human RA bone explants. Blocking of bone-derived endogenous IL-17A with specific inhibitors resulted in a protective inhibition of bone destruction (17, 44). A recent study confirmed that the combined blockade of TNFα and IL-17A is more effective in inhibiting cytokine, chemokine, and matrix enzyme responses from human mesenchymal cells and in blocking tissue destruction associated with a mouse model of arthritis (45).

In conclusion, TNF-α and IL-17A induced osteogenic differentiation of isolated FLS previously exposed to long-lasting disease related stress of OA and RA diseases. In general, isolated RA-FLS appeared more sensitive to the effects of TNF-α and IL-17A compared to OA-FLS. In RA bone explants where OBs interact with OCs and bone marrow cells, the addition of TNF-α and IL-17A induced bone matrix destruction and lower ALP production, indicating defective repair. In common OA, lower levels of these cytokines would allow some repair activity as reflected by osteophyte formation. From these findings, the combined blocking of TNF-α and IL-17A may represent a new option for controlling inflammatory bone disorders as in RA, psoriatic arthritis as well as in ankylosing spondylitis and inflammatory subsets of OA and even common osteoporosis.

## Conflict of Interest Statement

The authors declare that the research was conducted in the absence of any commercial or financial relationships that could be construed as a potential conflict of interest.

## Abbreviations

ALP, alkaline phosphatase; BMP-2, bone morphogenetic protein-2; FLS, fibroblast-like synoviocytes; OA, osteoarthritis; RA, rheumatoid arthritis; Runx2, runt-related transcription factor-2; Wnt5a, wingless-type MMTV integration site family, member 5A.
